# Expression of *λ* and K Genes Can Occur in all B Cells and is Initiated Around the Same Pre-B-Cell Developmental Stage

**DOI:** 10.1155/1994/87352

**Published:** 1994

**Authors:** Lynn Doglio, Joo Yeun Kim, Grazyna Bozek, Ursula Storb

**Affiliations:** 1 Department of Molecular Genetics and Cell Biology University of Chicago Chicago Illinois 60637 USA; 2 Department of Biochemistry Northwestern University Evanston Illinois USA

**Keywords:** K/λ isotypic exclusion, mouse genes, feedback inhibition of Ig-gene rearrangement

## Abstract

Transgenic mice that carry a λ2 transgene under the control of the Vλ2 promoter and the Eλ2-4 enhancer (λ2Eλ mice) are described. A high proportion of B cells in the spleen and the bone marrow express the λ  transgene on the cell membrane. λ2 protein is synthesized by all λ2Eλ-derived spleen B-cell hybridomas that have retained the transgene, suggesting that all B cells have the ability to express λ genes. Feedback inhibition of endogenous K-gene rearrangement is significant, but not complete. The results are similar to those with transgenic mice expressing the same λ2 transgene under the control of the heavy-chain enhancer (λ2EH mice). Although the λ2EH transgene is expressed before the λ2Eλ transgene, feedback inhibition seems to occur at about the same stage of B-cell development, regardless of the timing of expression of the λ transgenes. Apparently, feedback is not necessarily coincident with the assembly of a heavy-chain/light-chain complex in pre-B cells. Expression of λ in the normal fetal liver coincides with the expression of K; thus, it appears that λ-gene transcription is not delayed. The differential rearrangement of K and λ genes is discussed in the light of these findings.


*Developmental Immunology*, 1994, Vol 4, pp. 13–26
Reprints available directly from the publisher
Photocopying permitted by license only

© 1994 Harwood Academic Publishers GmbH
Printed in Singapore

### Expression of λ and κ Genes Can Occur in all B Cells and is Initiated Around the Same Pre-B-Cell Developmental Stage

LYNN DOGLIO,* JOO YEUN KIM, GRAZYNA BOZEK and URSULA STORB†

*Department of Molecular Genetics and Cell Biology, University of Chicago, Chicago, Illinois 60637*

Transgenic mice that carry a λ2 transgene under the control of the Vλ2 promoter and the Eλ2-4 enhancer (λ2Eλ mice) are described. A high proportion of B cells in the spleen and the bone marrow express the λ transgene on the cell membrane. λ2 protein is synthesized by all λ2Eλ-derived spleen B-cell hybridomas that have retained the transgene, suggesting that all B cells have the ability to express λ genes. Feedback inhibition of endogenous κ-gene rearrangement is significant, but not complete. The results are similar to those with transgenic mice expressing the same λ2 transgene under the control of the heavy-chain enhancer (λ2EH mice). Although the λ2EH transgene is expressed before the λ2Eλ transgene, feedback inhibition seems to occur at about the same stage of B-cell development, regardless of the timing of expression of the λ transgenes. Apparently, feedback is not necessarily coincident with the assembly of a heavy-chain/light-chain complex in pre-B cells. Expression of λ in the normal fetal liver coincides with the expression of κ; thus, it appears that λ-gene transcription is not delayed. The differential rearrangement of κ and λ genes is discussed in the light of these findings.

KEYWORDS: κ/λ isotypic exclusion; mouse λ genes; feedback inhibition of Ig-gene rearrangement.

### INTRODUCTION

Mammals produce both κ and λ light chains but in vastly different proportions. At one end of the spectrum, mouse serum Igs are about 95% $\kappa^+$. At the other end, horse Igs are 95% $\lambda^+$, whereas human light chains are divided between 60% $\kappa^+$ and 40% $\lambda^+$ (Eisen and Reilly, 1985). It is not clear how these ratios are regulated. Selection of $\kappa^+$ cells in the peripheral lymphoid organs only partially explains the skewing, because even in the bone marrow, $\kappa^+$ cells outnumber $\lambda^+$ cells by at least 5:1 (this paper; Rolink et al., 1993). Analysis of mouse and human Ig producing lymphomas and plasmacytomas has led to the hypothesis that κ and λ genes are sequentially activated (Hieter et al., 1981). It was found that most κ-producing B cells have their λ genes in germline configuration. In contrast, B cells with functional λ genes have, with few exceptions, both κ genes rearranged or even deleted. The sequential model postulated that developing B cells would first acquire the ability to rearrange κ genes. λ genes would be activated only after both κ genes had been nonproductively rearranged. A modification of this model is one in which the induction of λ-gene rearrangement requires the inactivation of the κ locus by rearrangement of the RS element located 3′ of the Cκ gene (Moore et al., 1985). Recently, the sequential model, in which λ rearrangement depends upon prior events at the κ locus, has essentially been ruled out by the analysis of κ knockout mice, which, despite the absence of any rearrangement in the κ locus, produce large numbers of B cells with rearranged λ genes (Takeda et al., 1993).

The ordered rearrangement of Ig genes is controlled by a V(D)J recombinase that is present in pro/preB cells until correct heavy- and light-chain gene rearrangements have occurred (reviewed in Storb et al., 1989). It was shown with κ transgenic mice, that B cells that express the κ transgene together with an endogenous heavy-chain gene

*Present address: Department of Biochemistry, Northwestern University, Evanston, Illinois.
†Corresponding author.

have their endogenous κ genes mostly unrearranged (Ritchie et al., 1984). This was taken as evidence for the shutoff of the V(D)J recombinase after completion of correct rearrangements of both a heavy-chain and a light-chain gene (Ritchie et al., 1984). Similarly, it has been shown in mice that express a λ transgene under the control of the heavy-chain enhancer that endogenous κ-gene rearrangement is strongly inhibited (Hagman et al., 1989; Neuberger et al., 1989). Thus, it was clear that λ, presumably the protein, can deliver a rearrangement feedback signal.

A puzzling observation was the finding of normal numbers of λ-producing B cells in κ transgenic mice, while endogenous κ-producing B cells were greatly diminished (Gollahon et al., 1988). This led to the hypothesis of two distinct B-cell populations with different abilities to regulate κ- and λ-gene expression. One, the κ lineage, was thought to rearrange only κ genes, whereas the other, the κ/λ lineage, was thought to be able to rearrange both κ and λ genes (Gollahon et al., 1988).

To test this model, it had to be determined whether λ genes can be expressed in all or in only a subset of B cells (Cuisinier et al., 1992). The enhancers driving λ-gene expression had for a long time been elusive, because it was found that, unlike heavy-chain and κ genes, no enhancers were present in the JC intron of λ genes (Picard and Schaffner 1984; Hagman et al. 1990). Recently, two strong B-cell-specific enhancers were found to be located 16 kb and 35 kb 3′ of the Cλ2-4 and Cλ3-1 constant-region gene clusters, respectively (Hagman et al., 1990). It was then possible to make transgenic mice with λ transgenes driven by the λ transcriptional control elements. We report here the analysis of transgenic mice with a rearranged λ2 transgene under the control of the Vλ2 promoter and the Eλ2-4 enhancer. They were generated to determine if all B cells can produce λ and to obtain additional clues concerning the control of the differential expression of κ and λ genes.

### RESULTS

### Tissue-Specific Expression

Expression of the λ2Eλ transgenes was determined by Northern blots of total RNAs from a variety of tissues. It was found that the λ2Eλ transgene was transcribed in the spleen, but not in thymus T cells (some λ mRNA was seen in thymus, but it was matched by κ mRNA from B-cell contamination), liver, kidney and heart, suggesting that expression is restricted to B cells (data not shown).

### FACS Analysis of Spleen Cells

To determine the distribution of $\lambda^+$ and $\kappa^+$ cells in the peripheral lymphoid organs, spleen cells of λ2 transgenic mice and normal littermates were analyzed by fluorescence-activated cell sorter (FACS). Total $Ig^+$ cells were determined by staining with anti-κ and anti-λ. The average of total lymphoid cells that are $Ig^+$ was $59.2 \pm 5.2$ for normal littermates, for λ2Eλ transgenics it was $52.6 \pm 4$ (λ94 line) and $36.6 \pm 4.8$ (λ96 line), and for λ2EH transgenics it was $51.7 \pm 0.7$.

Table 1 shows the percentages of λ and κ positive cells. Normal mice have on average only 14% total $\lambda^+$ cells, including half that are $\lambda^+/\kappa^+$. The majority, 93%, of the B cells are $\kappa^+$. In the λ2Eλ transgenic mice on the other hand, on average 68% (λ94) or 71% (λ96) of the B cells are $\lambda^+$. The majority of these coexpress κ (46% or 56%). Only 22% or 15% of the total B cells are λ-only cells, 32% or 29% are κ-only. Clearly, as has been found previously with λ2EH-gene transgenic mice (Hagman et al., 1989; Neuberger et al., 1989; Rudin et al., 1991), there is feedback on endogenous κ-gene rearrangement, but it is incomplete. No significant difference is seen between λ94 and λ96 λ2Eλ transgenic mice. The degree of feedback in the λ2Eλ mice seems very similar to that found in the λ2EH mice: Both show a decrease in total $\kappa^+$ cells and a substantial population of λ-only cells. However, in the mice with the λ2EH transgene more of the $\kappa^+$ cells coexpress λ (32% versus 16%). Perhaps this is a reflection of the somewhat greater amounts of λ protein on mature B cells of these mice due to greater efficiency of the heavy-chain enhancer at this stage. The total $\lambda^+$ B cells can be roughly divided into two populations based on staining intensity with anti-λ (not shown). In the λ2EH transgenic mice, 42% of the total $\lambda^+$ B cells express high levels of λ, however, only 31% of the $\lambda^+$ cells in λ2Eλ mice fall in this category.

Why are not 100% of the B cells $\lambda^+$? We assume that this is due to our inability to detect low levels of λ in many double-positive cells. This assumption is supported by the analysis of hybridomas.

TABLE 1
FACS Analysis of Spleen Lymphoid Cells

| Exp. no. | Mouse[a] | Age (wk) | % of Total $Ig^+$ spleen lymphoid cells[b] | | | | |
|---|---|---|---|---|---|---|---|
| | | | κ only | κ+λ | λ only | Total κ | Total λ |
| 1 | NLM-1 | 8 | 85 | 11 | 4 | 96 | 15 |
| | λ96-2 | 8 | 30 | 55 | 15 | 85 | 70 |
| | λ96-3 | 8 | 27 | 57 | 16 | 84 | 73 |
| | λEH-4 | 6 | 20 | 59 | 21 | 79 | 80 |
| | λEH-5 | 6 | 13 | 61 | 23 | 77 | 87 |
| 2 | NLM-2 | 6 | 88 | 3 | 7 | 91 | 10 |
| | NLM-3 | 6 | 87 | 9 | 4 | 96 | 13 |
| | NLM-6 | 6 | 86 | 5 | 9 | 91 | 14 |
| | NLM-7 | 6 | 86 | 7 | 7 | 93 | 14 |
| | λ94-1 | 6 | 34 | 46 | 20 | 80 | 65 |
| | λ94-4 | 6 | 35 | 43 | 22 | 78 | 65 |
| | λ94-5 | 6 | 34 | 43 | 23 | 77 | 66 |
| 3 | NLM-22 | 6 | 82 | 9 | 9 | 91 | 18 |
| | NLM-23 | 6 | 86 | 7 | 8 | 93 | 15 |
| | λ94-16 | 6 | 31 | 46 | 23 | 77 | 69 |
| | λ94-17 | 6 | 25 | 51 | 23 | 76 | 74 |

| | Mean % ± standard deviation | | | | |
|---|---|---|---|---|---|
| | κ only | κ/λ | λ only | Total κ | Total λ |
| NLM $n=7$ | 86.0 ± 2.0 | 7.0 ± 2.3 | 7.0 ± 1.8 | 93.0 ± 2.0 | 14.0 ± 2.6 |
| λ94 $n=5$ | 31.8 ± 4.0 | 45.8 ± 3.0 | 22.0 ± 1.3 | 77.6 ± 1.5 | 67.8 ± 3.8 |
| λ96 $n=2$ | 28.5 ± 2.1 | 56.0 ± 1.4 | 15.3 ± 0.4 | 84.5 ± 0.7 | 71.0 ± 1.4 |
| λEH $n=2$ | 16.0 ± 5.6 | 61.5 ± 3.5 | 22.0 ± 1.4 | 78.0 ± 1.4 | 83.5 ± 4.9 |

[a]Normal littermate (NLM) or transgenic mice (λ94 and λ96 are λ2Eλ mice; λEH are λ2EH mice).

[b]Total Ig determined by staining with anti-κ or anti-λ antibodies.

### Spleen B-Cell Hybridomas of λ2Eλ Transgenic Mice

Hybridomas were produced from the spleens of 4-week-old LPS-treated λ2Eλ transgenic mice. Of 67 hybridomas, 20 (30%) secreted κ, 33 (49%) secreted κ and λ, and 14 (21%) secreted λ only. These percentages are very similar to those of $Ig^+$ spleen cells determined by FACS analysis (Table 1). A subset of the hybridomas was further studied (Table 2). It was found that all the κ-only hybridomas had lost the transgenes. Similar results were found with hybridomas of λ2EH mice (Hagman el al., 1989). Based on these findings, it appears that the λ2 transgene under the control of its own enhancer can be expressed in all B cells. It is not clear whether most of the $κ^+$ cells in the spleen (as determined by FACS analysis) have also lost the transgene. The fact that the percentages of κ-only cells in spleen and hybridomas agree so well may suggest that this is the case. However, hybridomas are notorious for the loss of chromosomes due to their polyploidy. It appears more likely that the "κ-only" B cells in the spleen express the λ transgene, but that κ has a higher affinity for many heavy chains. It has been shown that in a competitive situation, even a slightly higher affinity of one type of light chain with the heavy chain results in its preferential association with the heavy chain (Grey and Mannik, 1965; Margulies et al., 1976; Klein et al., 1979).

All λ-only hybridomas show only germline κ genes. Of course, it cannot be ruled out that some of them have lost a chromosome carrying a rearranged κ gene. However, in normal mice, only 60% of the $κ^+$ cells have retained one germline allele and essentially none of the $λ^+$ cells retain a germline κ allele (Coleclough et al., 1981). Thus, the λ hybridomas from these λ2Eλ mice support the conclusion from the FACS analysis that the λ2Eλ transgene causes feedback inhibition of κ-gene rearrangement.

On the other hand, only 30% of the double $κ^+/λ^+$ hybridomas and none of the κ hybridomas show a germline κ allele. Presumably, the $κ^+$ cells that have a germline κ allele had produced a functional κ allele by rearrangement before expression of the λ

TABLE 2

Hybridomas of 4-week-old λ2Eλ-94 Transgenic Mice

| Hybridoma no. | ELISA[a] | Transgene[b] | κ genes[c] | JH 3,4[d] | 5′DH[e] | 5′JH[f] | H genes[g] |
|---|---|---|---|---|---|---|---|
| 5-2 | D | + | R | G | 1B | – | VDJ/DJ |
| 5-3 | D | + | R | G | 1B | – | VDJ/DJ |
| 5-13 | D | + | G/R | G | G | G | VDJ/G |
| 5-17 | D | + | – | G | G | G | VDJ/G |
| 5-18 | D | + | R/R | – | 1B | – | VDJ/DJ |
| 5-20 | D | + | G/R | G | 3B | – | VDJ/DJ |
| 5-40 | D | + | G/R | G | 3B | – | VDJ/DJ |
| 5-45 | D | + | – | G | 1B | – | VDJ/DJ |
| 5-52 | D | + | – | – | 1B | – | VDJ/DJ |
| 5-55 | D | + | R | R | 1B | – | VDJ/DJ |
| 5-31 | K | – | R | – | – | – | VDJ |
| 5-39 | K | – | – | G | G | G | VDJ/G |
| 5-43 | K | – | R | – | – | – | VDJ |
| 5-49 | K | – | R | – | – | – | VDJ |
| 5-51 | K | – | R | G | – | – | VDJ |
| 5-54 | K | – | R | – | – | – | VDJ |
| 5-6 | L | + | G | – | 1B | – | VDJ/DJ |
| 5-7 | L | + | G | – | 1B | – | VDJ/DJ |
| 5-10 | L | + | G | G | G | G | G |
| 5-11 | L | + | G | G | G | G | G |
| 5-14 | L | + | G | G | 1B | G | G/DJ |
| 5-25 | L | + | G | G | 1B | – | VDJ/DJ |
| 5-26 | L | + | G | G | G | G | G |
| 5-38 | L | + | G | G | G | G | G |
| 5-41 | L | + | G | G | G | G | G |
| 5-44 | L | + | G | G | G | G | G |
| 5-48 | L | + | G | G | G | G | G |
| 5-50 | L | + | G | G | G | G | G |
| 5-59 | L | + | G | G | G | G | G |
| 5-60 | L | + | G | G | 3B | – | VDJ/DJ |

[a]ELISA of the hybridoma secretions showed κ and λ(D), κ(K), or λ (L) protein.

[b]Presence of the λ transgene was determined by Southern blot.

[c]Rearrangement of endogenous κ genes was determined from Southern blots of Bam HI-digested DNA probed first with a $C_\kappa$ probe and after stripping reprobed with a 5′ of Jκ1 probe. R = one rearranged κ gene. G=germline κ gene. –=κ gene is not present.

[d]H genes were analyzed by digestion with Bam HI and probing with a JH 3-4 probe (Manz et al., 1988). G=germline band; R=rearranged band; –=no band detected

[e]Digestion with EcoRI and probing with a 5′ DH probe (Manz et al., 1988) shows five bands if no rearrangement has occurred (=G). DJ rearrangement generally leads to reduction of the number of bands (1B, 3B=1 or 3 bands). –=VD rearrangement deletes all bands with this probe.

[f]Digestion with EcoRI and probing with 5′ JH (Manz et al., 1988) results in a band only if no DJ rearrangement has occurred (G).

[g]Conclusion concerning the H-gene status. VDJ=heavy chains are secreted. DJ=at least one band is seen with the 5′ DH probe. G=band with the 5′ JH probe.

transgene led to feedback inhibition of the V(D)J recombinase. Because the κ-only and double κ/λ hybridomas have fewer germline κ alleles than κ$^+$ cells from normal mice, the parent B cells must have been selectively enriched, presumably by antigen.

The presence of a band in Southern blots of DNA hybridizing with a probe derived from a region 5′ of JH1 indicates the presence of an unrearranged heavy-chain allele (Alt et al., 1984). Many of the hybridomas showed evidence of such a germline H allele (Table 2). This was confirmed by the presence of germline bands when the blots were probed with a sequence from 5′ of DH (Table 2). The proportion of hybridomas with germline heavy-chain alleles is similar to that of hybridomas from λ2EH transgenic mice (Hagman et al., 1989). Germline heavy-chain alleles are very rare in hybridomas from normal mice (Roth et al., 1993; Weaver et al., 1985). In transgenic hybridomas that produce a heavy chain, the presence of a germline H locus presumably indicates that the productive heavy-chain gene rearrangement occurred in a pre-B cell already expressing the light-chain transgene and that the V(D)J recombinase was inactivated before the second heavy-chain allele could begin to rearrange.

Most of the λ-only hybridomas show only germline heavy-chain genes with no evidence of any heavy-chain gene rearrangement. Although many of these may have lost one chromosome 12 carrying the rearranged heavy-chain allele, it seems unlikely that all of them did, particularly, given that all the κ$^+$ and double-producing hybridomas show at least one rearranegd heavy-chain allele. It appears then that these mice may have some pre-B cells in the spleen and that these cells can be readily fused with myeloma cells. To a lesser degree, a similar finding was made with hybridomas from λ2EH mice (Hagman et al., 1989). It is puzzling that in FACS analysis, many more λ-only B cells were found than $\lambda^+$ hybridomas that have heavy chains. This has not been further explored.

### FACS Analysis of Bone Marrow B Cells

Because spleen B cells represent a population of antigen-selected cells, we analyzed bone marrow cells in order to evaluate the distribution of κ and λ cells that are largely unselected (Table 3). 33.9 to 51% of the total lymphoid cells in the bone marrow were $Ig^+$. The averages were $43.5 \pm 4.5$ for normal littermates, $47.2 \pm 4.5$ (λ94) and $34.3 \pm 0.6$ (λ96) for λ2Eλ transgenics, and $40.6 \pm 1.5$ for λ2EH transgenic mice. 17% of $Ig^+$ bone marrow B cells of normal littermates express λ. This is slightly higher than in spleen and persumably more closely reflects the actual rate of λ rearrangements in the bone marrow as described before (Kim et al., 1994).

In the λ transgenic mice, an average of 51 to 69% of the bone marrow B cells are $\lambda^+$ (Table 3). In contrast to the spleen (Table 1), in the bone marrow of λ transgenic mice, more than 50% of $\lambda^+$ cells express λ only. However, the λ transgenic mice also have a higher percentage of κ-only versus κ/λ B cells. Does this mean that the endogenous κ enhancer is active before the transgenic λ enhancer? This is unlikely given findings with fetal liver (see what follows). Also in λ2EH transgenic mice, κ-only B cells exceed the number of κ/λ B cells. The basis of these findings is not clear. Overall, similar proportions of κ and λ positive cells are found at 6 weeks of age in the bone marrow of both λ2Eλ and λ2EH mice.

Compared with normal littermates, in λ transgenic mice, regardless of the type of enhancer present in the transgenes, the strongest transgene-induced effect in bone marrow cells is a reduction by over 50% of the number of $B220^{high}/\kappa^+$ lymphoid cells (Fig. 1). Thus, both types of transgenes induce feedback inhibition of endogenous κ-gene rearrangement, although the inhibition seems stronger with the λ2EH transgene (Fig. 1B). However, $B220^{low}/\kappa^+$ cells are present in very similar numbers in normal and both types of λ transgenic mice. The

TABLE 3
FACS Analysis of Bone Marrow Lymphoid Cells

| Exp. no. | Mouse[a] | Age (wk) | % of Total Ig+ Cells | | | | |
|---|---|---|---|---|---|---|---|
| | | | κ only | κ/λ | λ only | Total κ | Total λ |
| 1 | NLM-1 | 8 | 91 | 3 | 6 | 94 | 9 |
| | λ96-2 | 8 | 50 | 25 | 24 | 75 | 49 |
| | λ96-3 | 8 | 48 | 24 | 28 | 72 | 52 |
| | λEH-4 | 6 | 31 | 18 | 51 | 49 | 69 |
| | λEH-5 | 6 | 29 | 19 | 52 | 48 | 71 |
| 2 | NLM-2 | 6 | 81 | 4 | 15 | 85 | 19 |
| | NLM-3 | 6 | 87 | 5 | 8 | 92 | 13 |
| | NLM-6 | 6 | 74 | 14 | 12 | 89 | 26 |
| | λ94-1 | 6 | 27 | 26 | 46 | 53 | 72 |
| | λ94-4 | 6 | 28 | 21 | 51 | 49 | 72 |
| | λ94-5 | 6 | 23 | 21 | 55 | 44 | 76 |
| 3 | NLM-22 | 6 | 87 | 3 | 10 | 90 | 13 |
| | NLM-23 | 6 | 87 | 4 | 8 | 91 | 13 |
| | λ94-16 | 6 | 32 | 25 | 42 | 57 | 67 |
| | λ94-17 | 6 | ~35 | ~24 | 36 | 59 | 60 |
| | | | Mean % ± standard deviation | | | | |
| | | | κ only | κ/λ | λ only | Total κ | Total λ |
| | NLM $n$=6 | | 83.2 ± 5.8 | 6.0 ± 4.5 | 10.6 ± 2.9 | 89.2 ± 2.8 | 16.8 ± 5.7 |
| | λ94 n=5 | | 29.0 ± 4.6 | 23.4 ± 2.3 | 46.0 ± 7.4 | 52.8 ± 5.7 | 68.8 ± 7.7 |
| | λ96 $n$=2 | | 49.0 ± 1.4 | 24.5 ± 0.7 | 26.0 ± 2.8 | 73.5 ± 2.1 | 51.0 ± 1.4 |
| | λEH $n$=2 | | 30.0 ± 1.4 | 18.5 ± 0.7 | 51.5 ± 0.7 | 48.7 ± 0.7 | 69.0 ± 2.8 |

[a]Normal littermate (NLM) or transgenic mice (λ94 and λ96 are λ2Eλ mice; λEH are λ2EH mice).

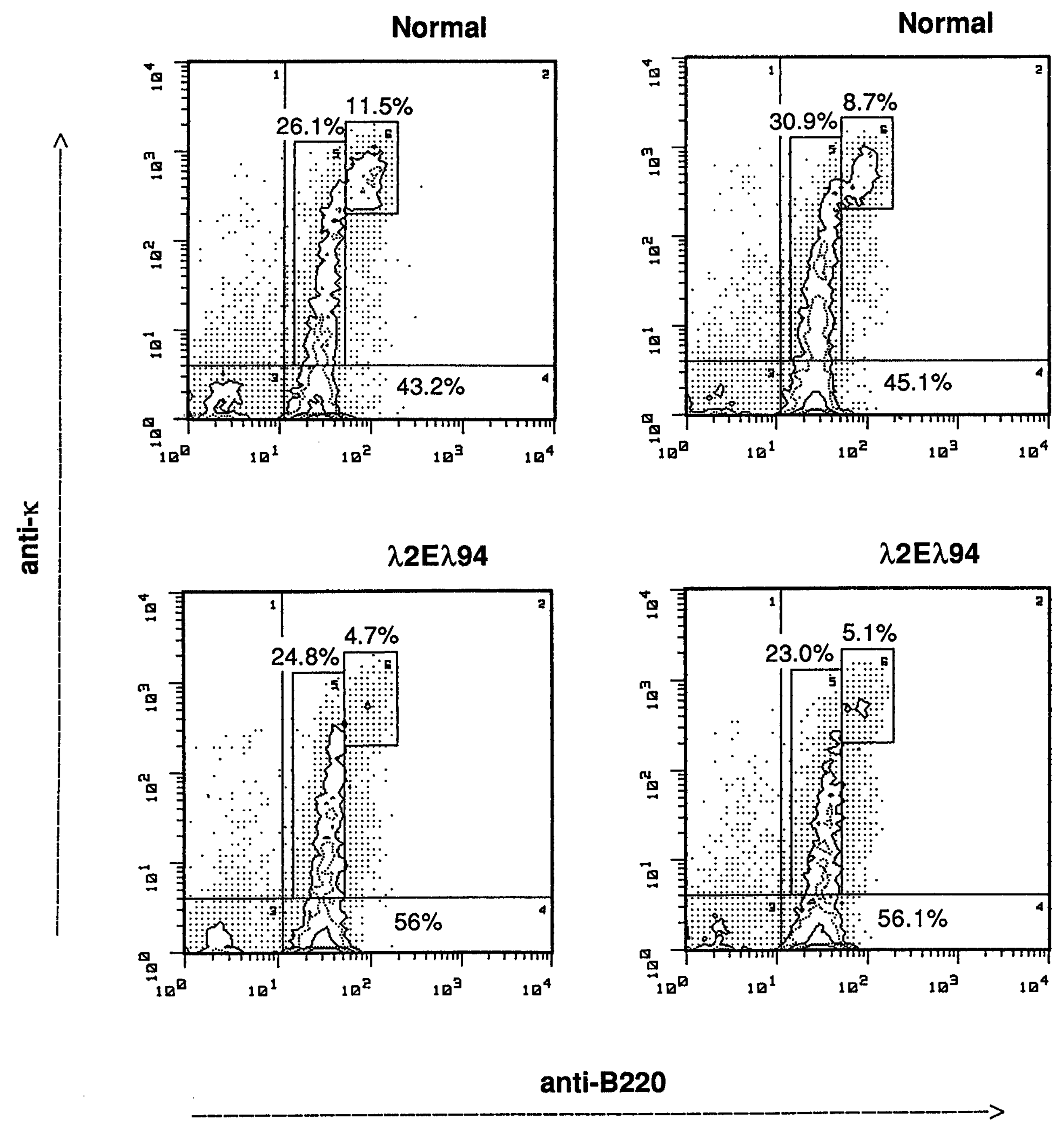

FIGURE 1A. FACS analysis of bone marrow lymphoid cells. (A) and (B) are from different experiments.

reduction in κ$^+$ cells occurs, therefore, at the transition from B220$^{low}$ to B220$^{high}$ B cells.

### Kappa and Lambda and mRNAs in Fetal Livers

In order to more directly determine the timing of expression of the λ transgenes, fetal liver RNAs were analyzed (Fig. 2). Expression of the λ2EH transgene is detectable in Northern blots in day 14 fetal livers and reaches a peak at day 15. Expression of the λ2Eλ transgenes is seen at day 15 and reaches a plateau at day 16 in Northern blots of fetal liver RNA of λ96 mice (Fig. 2A). In Northern blots of RNA from λ94 mice, the mature λ2 mRNA signal is

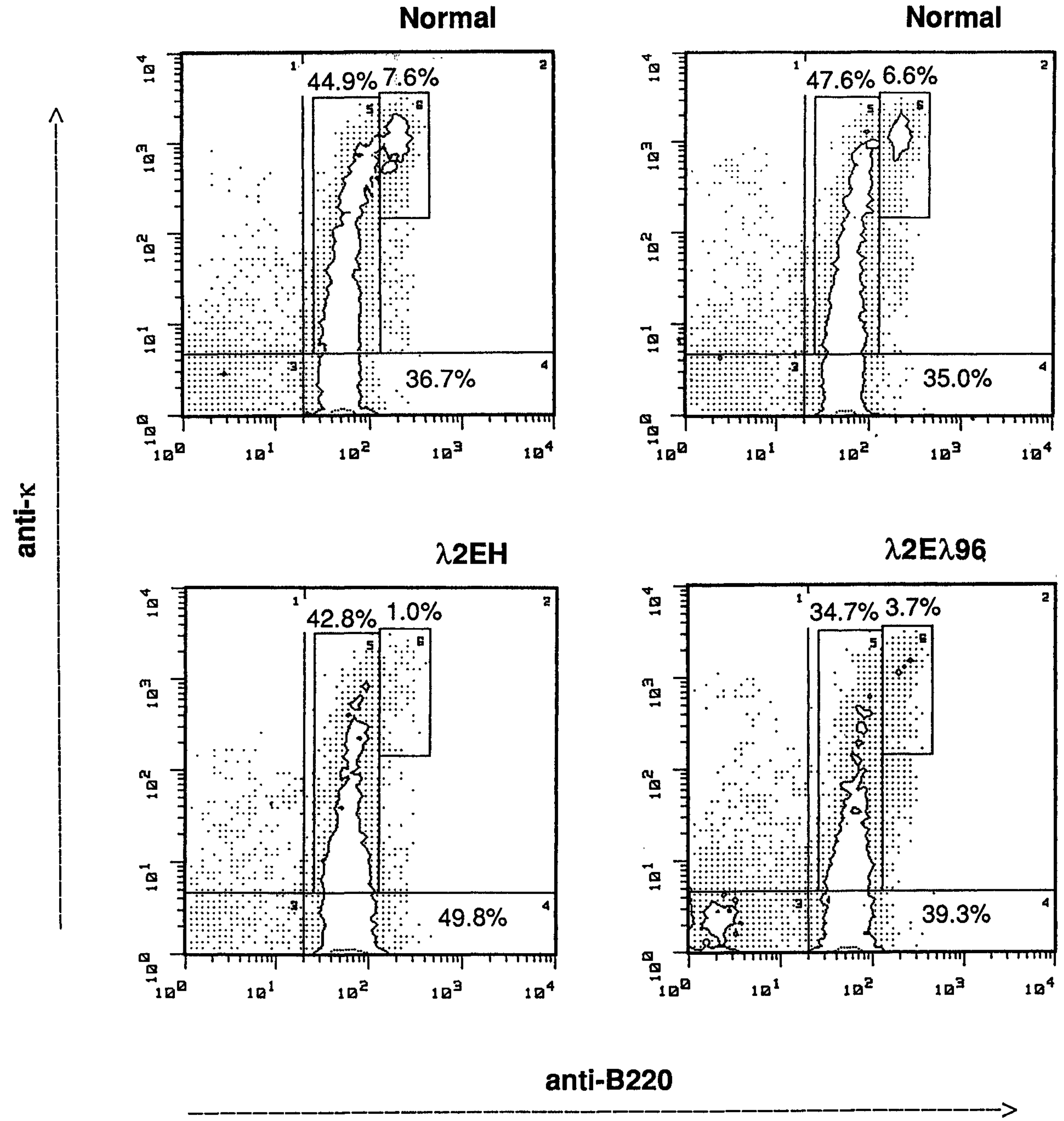

FIGURE 1B. FACS analysis of bone marrow lymphoid cells. (A) and (B) are from different experiments.

not seen until day 16, at which time peak levels are present also in this mouse (Fig. 2A). This expression pattern is confirmed by PCR of fetal liver RNAs from λ94 mice (data not shown). Presumably, the overall levels are lower in λ94 compared with λ96 due to the lower-transgene copy number. However, the time course seems to be the same in both λ2Eλ transgenic lines with peak levels being delayed by about 1 day compared with the λ2EH transgenic line. There seems to be a decline of λ RNA levels for both λ2Eλ transgenic lines at day 18, but not for the λ2EH line. Perhaps this is due to limiting amounts of transcription factors shared between κ and λ genes, such as PU.1 (Eisenbeis et al., 1993). Com-

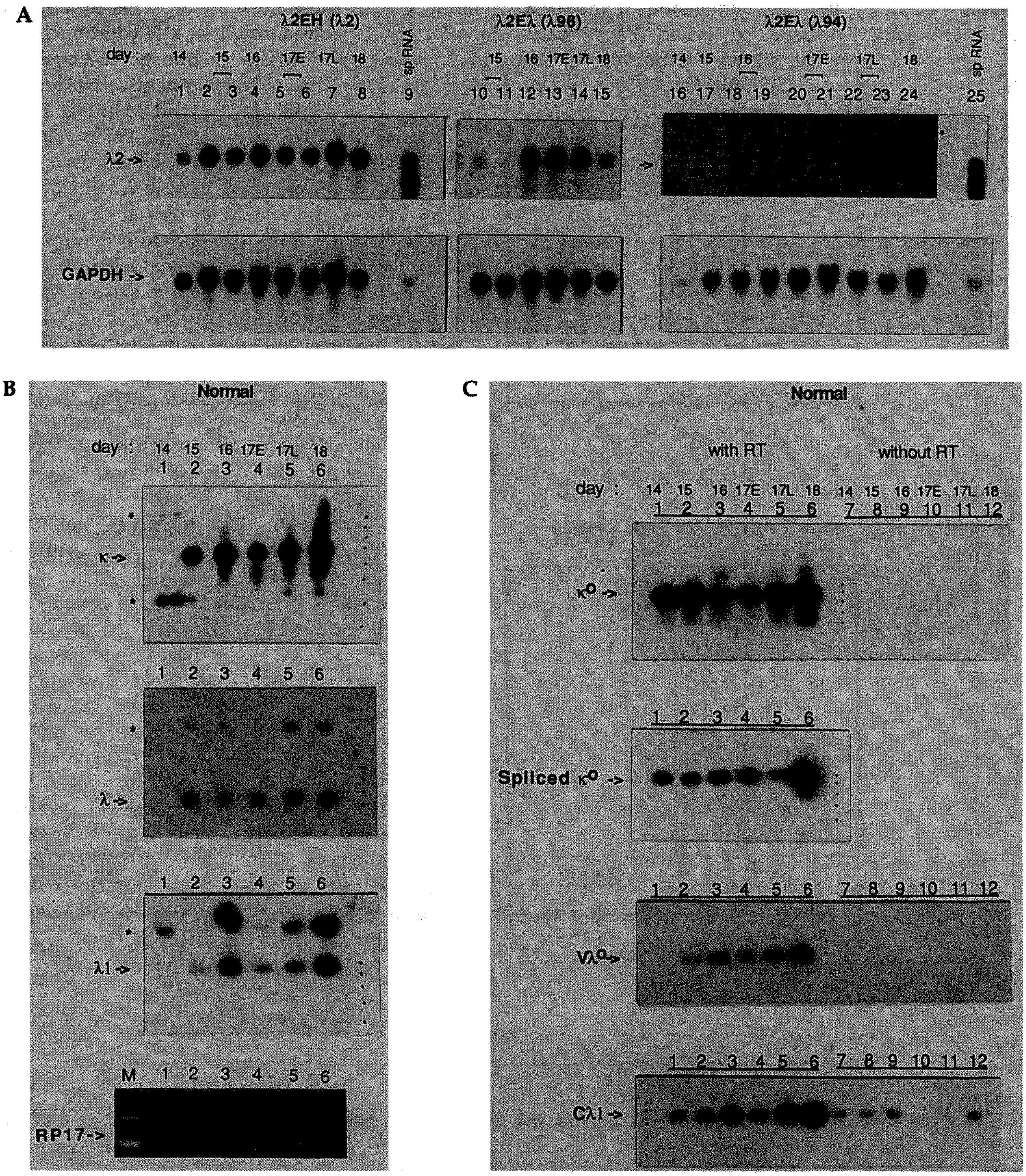

FIGURE 2. Analysis of λ and κ mRNAs in fetal liver of transgenic and normal mice. (A) Northern blots of fetal liver RNAs from λ2EH and λ2Eλ transgenic fetuses: top, λ2; bottom, GAPDH. (B) PCR analysis of mature κ; λ1, 2, 3 (λ); and λ1 mRNAs, as well as ribosomal protein-17 mRNA of normal fetal livers. The mature transcripts are indicated by an arrow. Asterisks are unknown bands that may represent DNA or unspliced RNAs or priming in unrelated RNAs. The same two samples are shown for day 14 in the κ panel, with no blank lane in between. (C) PCR analysis of sterile transcripts in the same fetal liver RNAs as in (B). Reactions with reverse transcriptase are shown on the left, and without reverse transcriptase on the right for the three assays ($\kappa^0$, $V\lambda^0$, and Cλ1) in which the amplified RNA transcripts are of the same size as amplified DNA. There is a background level of Cλ1 amplification without reverse transcriptase, but the level with reverse transcriptase well exceeds it. The days of gestation are indicated (17 E, L = day 17 early, late).

petition may exist between endogenous κ and/or λ gene expression and the expression of the λ2Eλ transgenes. Because this seems not the case for the heavy-chain enhancer, the latter factors may not be limiting in the developing B cells.

To compare the timing of expression of endogenous κ and λ genes, fetal liver RNA from normal mice was analyzed by PCR, because Northern blots were found not to be sensitive enough. No evidence for the expression of rearranged κ and λ genes was obtained at day 14, but the mature forms of both κ and λ mRNAs were clearly present at day 15 (Fig. 2B). The λ mRNAs were determined using Vλ and Cλ primers, which detect all three forms of λmRNA (V2JC2, V1JC3, and V1JC1; see Materials and Methods). A similar profile of expression was seen with V1JC1 specific primers (Fig. 2B). These data suggest that competency for rearrangement and expression of κ and λ genes arise in parallel.

To further compare the activation of the κ and λ loci in developing B cells, sterile transcripts from the two loci were analyzed (Fig 2C). Both $\kappa^0$ and Cλ transcripts were seen at day 14. The Cλ transcripts could be either germline or mature. However, because no mature λ transcripts were found at day 14, these must be sterile. Sterile Cλ transcripts have also been seen in rat (Hellman et al., 1985). A spliced $\kappa^0$ transcript was also detectable at day 14. The latter had previously been reported only in Abelson murine leukemia virus transformed pre-B cell lines (Leclercq et al., 1989), but, as shown here, is also present in normal pre-B cells. A sterile Vλ transcript was not detectable until day 15 (Fig. 2C). Thus, it seems that the appearance of mature λ mRNA and presumably rearrangement of λ genes does not occur until the Vλ promoter is active. This cannot be tested for κ because no consensus sequence exists of the region 3′ of Vκ genes.

### DISCUSSION

The λ2Eλ transgenes are expressed tissue specifically, namely, only in B cells. They are thus similar in expression to λ1 transgenes that contained a similar length of sequence upstream of the Vλ promoter, but included the complete sequences between Cλ1 and the Eλ3-1 enhancer (about 35 kb) and additional 10 kb 3′ of the enhancer (Eccles et al., 1990).

It appears from the findings with the λ2Eλ transgenic mice that λ genes can be expressed in most, if not all B cells. Between 65% and 74% of spleen B lymphocytes in these mice are $\lambda^+$. This proportion must be higher, considering that κ/λ coproducing B cells may often not express the transgenic λ chain in membrane Ig because of the competitive binding of κ with the heavy chain (Grey and Mannik, 1965; Margulies et al., 1976; Klein et al., 1979). This is confirmed by spleen B-cell hybridomas containing the λ transgene all of which secrete λ light chains (24/24). These results suggest that all B cells are capable of λ expression. The data do not support a model of a separate κ B-cell lineage (Gollahon et al., 1988). The implications for isotypic exclusion of κ and λ genes will be discussed below.

The λ transgenes containing the λ enhancer lead to significant feedback inhibition of κ-gene rearrangement, very similar to that observed with the λ2EH transgene (Tables 1 and 3; Hagman et al., 1989). The percentage of newly synthesized $\kappa^+$ B cells in the bone marrow is not significantly lower in the λ2EH transgenic mice compared with the λ2Eλ mice. It is expected, and borne out by the RNA analysis of fetal liver, that the λ transgene with the heavy-chain enhancer is expressed earlier than the one with the λ enhancer, namely, at the time of the earliest H-gene expression. One might have expected to see much stronger feedback on κ-gene rearrangement by the λ2EH transgene, because the transgenic light chain could associate with heavy chains as soon as a functional heavy-chain gene is created. The actual finding may indicate that feedback inhibition does not occur automatically after heavy-chain/light-chain assembly. Rather, the pre-B cells may have to reach a special stage in their development and/or physically move to a particular stromal cells compartment before feedback can ensue. The physiological stimulus for the feedback is unknown, but in vitro experiments with N-myc pre-B cells have suggested that the shutoff of the V(D)J recombinase may require crosslinking of the B-cell receptor (Ma et al., 1992). A similar conclusion was drawn from studies with cultured pre-B cells (Rolink et al., 1993) and with pre-T cells (Turka et al., 1991).

The suggestion of delayed feedback is further supported by the analysis of bone marrow B cells expressing κ and B220 (Fig. 1). In λ transgenic mice with either the λ or heavy-chain enhancer, the percentages of $B220^{low}/\kappa^+$ cells are about the same as in normal mice. However, the $B220^{high}/\kappa^+$ cells are reduced by 50% or more. This may indicate that κ rearrangement continues during the $B220^{low}$ stage and is only inhibited at the time when B cells that have productively rearranged a κ gene transit into the B220$^{high}$ stage. In that way, a large proportion of newly arising B cells would coexpress endogenous κ and transgenic λ. Such a delayed feedback may explain the production of endogenous light chains replacing anti-self light chains in what has been termed "receptor editing" (Tiegs et al., 1993). It would also explain the escape of endogenous L chain producing B cells from feedback inhibition by rearranged κ or λ transgenes (Manz et al., 1988; Neuberger et al., 1989; Bogen and Weiss, 1991; Rudin et al., 1991).

The development of expression of Ig genes in fetal liver roughly parallels that of pre-B cells in the bone marrow, but represents a synchronized population of pre-B cells (Strasser et al., 1989). It is clear from the Northern analysis that the expression of the λ2EH transgene precedes that of the λ2Eλ transgene (Fig 2). Expression of the former, being regulated by the heavy-chain enhancer, presumably is simultaneous with μ-gene expression in fetal mice (Alt et al., 1981). Endogenous κ- and λ- gene expression is seen as sterile transcripts as early as day 14. Furthermore, mature κ and λ transcripts indicative of gene rearrangement are seen by day 15 of gestation. There is therefore no indication that the expression of κ precedes that of λ.

If all B cells have the ability to rearrange both classes of light-chain genes at the same time, why then are less than 20% of the B cells arising in the normal bone marrow expressing a λ gene? Because the findings in κ-knockout mice essentially refute a model that explains κ/λ isotypic exclusion by making λ rearrangement dependent on a signal from the κ locus, it appears that the chance of rearrangement for λ is simply lower than for κ. Stochastic models have been considered a number of times (Coleclough, 1983); the question remains why λ has a lower probability for rearrangement. The timing of the expression of κ and λ genes seems to be similar as shown by the findings with fetal liver RNAs (Fig 2). Additionally, pre-B cell lines have been found to express reporter genes under the control of the λ enhancer (C. Rudin, B. Kurtz and U.S., unpublished). However, although λ appears to be initially expressed at the same time as κ, the strength of activation of the κ and λ enhancers may be different. The κ gene has two enhancers, whereas, due to spatial constraints (Storb et al., 1989; Hagman et al., 1990), each λ gene may be governed by only one of the two λ enhancers. Unlike the λ enhancers, the two κ enhancers are controlled by different transactivating factors. Only the κ intron enhancer is activated by NFκB. Interestingly, the λ enhancers have a certain homology with the 3′ κ enhancer (Pongubala et al., 1992; Eisenbeis et al., 1993). Thus, the λ enhancers may compete with the 3′ κ enhancer for the binding of the same transactivating factors and the 3′ κ enhancer may have a higher binding affinity. To completely unravel the isotypic exclusion of κ and λ, the activation and relative strengths of the κ and λ enhancers must be more systematically analyzed once the transactivating proteins are fully understood.

Another possibility to explain the κ/λ imbalance is that the κ and λ genes represent unequal targets for the V(D)J recombinase (Miller et al., 1982). It has been reported that in an in vitro system using rearrangement constructs containing the κ and λ rearrangement signal sequences (RSS), the frequency of κ rearrangements is higher (Ramsden and Wu, 1991). However, because rearrangement via the λ RSSs in this report was unphysiologically low (two to three orders of magnitude lower than that via the κ RSSs), this claim must be evaluated by additional experiments.

An important difference between the two loci are the numbers of RSSs in V and J genes that can serve as targets for rearrangement. The κ locus has at least 50 V genes and 4 functional J genes (Tonegawa, 1983). The λ locus has only three V genes (V1, V2, and Vx) and three functional J genes (J1, J2, and J3) (Miller et al., 1988; Carson and Wu, 1989; Storb et al., 1989). Thus, if the levels of V(D)J recombinase in pre-B cells are limiting, κ genes will have a much greater chance for encounter with recombinase and therefore rearrangement. Recent data with transgenic κ-rearrangement substrates have ruled out a mechanism in which the V(D)J recombinase would bind to a single RSS as a holo-enzyme complex with binding sites for both the 12-spacer RSS and the 23-spacer RSS and track to the nearest complementary RSS (Engler et al., 1993). The data are most compatible with binding and random encounter of at least two independent subunits, one with specificity for a 12-spacer RSS and the other for a 23-spacer RSS in a situation where the recombinase is present in a limiting quantity. Nonsaturating levels of the recombinase, at least at the stage of L-gene rearrangement, are also supported by the finding that about 60% of κ$^{+}$ B cells have one κ allele in germline conformation (Coleclough et al., 1981). In this way, the problem of unequal levels of κ and λ expression would be reduced to unequal numbers of recombinase targets. The suggestion of limiting recombinase levels will have to be tested.

Finally, the possibility of λ and κ locus-specific rearrangement coactivators (or silencers; Lauster et al., 1993) that may exist in unequal concentrations must be considered in future experiments.

### MATERIALS AND METHODS

### λ2 Transgenes

Construction of the λ2 transgene containing the H-chain enhancer (λ2EH) and the transgenic line carrying this transgene (λ2-1275) have been described (Hagman et al., 1989). A λ2 transgene driven by the Eλ2-4 enhancer was constructed as follows (Fig. 3): The 1.6-kb XhoI-SalI fragment containing the λ2 enhancer was isolated from pA16-N16 (Hagman et al., 1990). This A16XS1.6 fragment was then subcloned into the SalI site of λ2-4.4X, which contains the functionary rearranged λ2 gene of the plamacytoma MOPC 315 (Wu et al., 1982; Hagman, 1989; Hagman et al., 1989). The resulting plasmid, λ2Eλ, was digested with SalI and PvuI to remove most of the vector, the insert was isolated from Sea Plaque (FMC Corp., Rockland, ME) low-melting agarose gels, and microinjected into the pronucleus of C57BL/6J zygotes.

### Transgenic Mice

Two λ2Eλ transgenic lines were chosen for analysis. One line, λ2Eλ-94, has 8–10 copies of the transgene, and the other line, λ2Eλ-96, has approximately 50 copies.

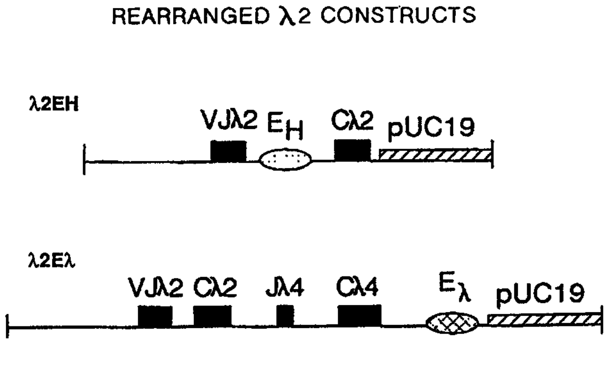

FIGURE 3. The lambda transgenes. Top, λ2EH; bottom, λ2Eλ.

### Hybridomas

λ2Eλ-94 mice were injected with 20 μg of LPS intraperitoneally 3 days prior to fusion. Hybridomas were generated as previously described (Manz et al., 1988). Supernatants were screened for Ig L chains in an ELISA assay, as described in detail elsewhere (Hagman et al., 1989). Briefly, for kappa production, goat anti-mouse (GAM)Ig (1:400; Kirkegaard & Perry Laboratories, Gaithersburg, MD) was the solid-phase antibody followed by peroxidase-conjugated GAMκ (1:1000; Southern Biotechnology Assoc., Birmingham, AL). Lambda L-chains were detected using a combination of GAMλ and peroxidase-conjugated GAMλ (1/200 and 1/1000, respectively; Southern Biotechnology Assoc.). In both assays, the TMB Microwell Peroxidase Substrate System (Kirkegaard & Perry Laboratories) was used as the substrate. The reaction was stopped with 1 M phosphoric acid and the absorbance was read at 450 nm.

### Nucleic Acid Procedures and Probes

DNA isolated from hybridomas or fetal tissue was phenol/chloroform extracted using standard techniques. Thirty micrograms of hybridoma DNA was digested with Bam HI or Eco RI (New England BioLabs, Beverly, MA), electrophoresed and blotted to Gene Screen Plus (New England Nuclear, Boston). Hydridization and reprobing procedures have been described (Manz et al., 1988).

RNA was isolated from the fetal liver of individual embryos at 14 to 18 days gestation according to the method of Chomczynski and Sacchi (1987). Body DNA was also isolated to detemine which embryos were transgenic.

Probes for Cκ, pX2.1 $J_H4$, 5′ of $J_H1$, pDFL-2.7 (Manz et al., 1988) and Cλ2 (Hagman et al., 1989) have been described previously.

### RNA Analysis by Northern Blots

Twenty micrograms of fetal liver RNA or 5 μg of normal spleen RNA, as control, were analyzed by Northern blot hybridization as described (Hagman et al., 1989). λ2 mRNA was detected by a $^{32}$P-labeled Cλ2 probe (Hagman et al., 1989). Under the same condition, there was no detectable signal of endogenous λ2/3 transcripts from nontransgenic fetal liver RNA (not shown). The blots were reprobed with a glyceraldehyde-3-phosphate dehydrogenase (GAPDH) probe (Fort et al., 1985).

### RNA PCR Analysis

The first strand of cDNA was synthesized from 5 μg of total RNA using Avian Moloney Leukemia Virus reverse transcriptase (15 U, Boehringer Mannheim) in 20 μl of reaction mixture containing 50 mM Tris, pH 8.5, 8 mM $MgCl_2$, 30 mM KCl, 1 mM DTT, 0.2 mM dNTPs (Pharmacia), RNasin (1 U/μl, Promega), random hexamer primer (20 pmoles, Pharmacia). The reaction was carried out at 42°C for 2 hours. After heat denaturation at 65°C for 15 min, 30 μl of TE was added to the reaction mixture. Ten microliters of the first-strand cDNA was amplified for mature κ or λ transcripts, and 1 μl of the cDNA for the control transcript (ribosomal protein-17 mRNA) using the following primers: Vκ degenerate primer and Cκ3 primer for κ transcripts (product size of 695 bp), Vλ1 and Cλ2 primers for λ transcripts (218 bp), Vλ1 and Cλ13 for λ1 transcripts (638 bp), RP17 primers for control transcripts (170 bp). For the sterile transcripts that do not span introns, total RNA was treated with RNase-free DNase (Promega). Five micrograms of DNase-treated RNA was used for the the cDNA synthesis as described earlier, with and without reverse transcriptase in parallel to make sure that the amplification occurs from the cDNA templates. The following primer pairs were used: Ko and Jκ2 primers for the sterile κ transcripts (product size of 500 bp), Ko and Cκ3 for the spliced $\kappa^0$ transcripts (681 bp), Vλ1 and 3Vλ1 primers for the Vλ sterile transcripts (262 bp), Cλ15 and Cλ13 for Cλ1 transcripts (466 bp).

The PCR reaction mixture contained Taq buffer (50 mM KCl, 10mM Tris, pH 8.3, 1.5 mM $MgCl_2$, 0.01% (w/v) gelatin), 0.2 mM dNTPs, 1 U Taq polymerase, 25 pmoles of primers, and cDNA template in a volume of 25 μl. Amplification was performed using a Perkin Elmer Cetus GenesAmp PCR System 9600 programmed for 30 cycles (28 cycles for the control transcript within the linear range of amplification) of thermal denaturation (at 95°C for 30 s), primer annealing (at 65°C for 30 s), and template extension (at 72°C for 30 s). Ten microliters of the amplified products were analyzed by Southern blot hybridization using specific probes: Cκ probe for mature κ transcripts, Jκ2 probe (Lewis et al., 1982) for sterile κ transcripts, Vλ1 probe for mature λ and sterile Vλ transcripts, and Cλ1 probe for Cλ1 transcripts.

The primers used were as follows: Vκ degenerate and Ko primers were described by Schlissel and Baltimore (1989); Cκ3 (3′ of poly A signal): 5′-ACAGAGATCTCAAGTGCAAAGACTC-3′; Vλ1 (within hv2): 5′-AACCGAGCTCCAGGTGTTCCT-GCCAGATT-3′; 3Vλ1 (60 bp 3′ of coding sequences): 5′-CCAAGCTTATGTAGCCACCTGTT-AAGAAAGTGGTAG-3′; Cλ15 (5′ end of coding sequences): 5′-GCAAGCTTAAGTCTTCGCCAT-CAGTCACCCTGTTTCC-3′; Cλ13 (3′ of poly A signal): 5′-CTATTCTAGAATAACAGGTTGTGAA-TGAAC-3′; Cλ2 (5′-GTGGACTTGGGCTGACC-TGTG-3′, the Cλ2 primer has homology to all three Cλ sequences, but anneals preferentially to Cλ2/3); RP17 upstream primer: 5′-TTTTACCAAGGACCC-GCCAACATG-3′; RP17 downstream primer: 5′-CTATCTTGTTGCGGAGCTTTTTGC-3′.

### Flow Cytometry

Spleen and bone marrow cells were stained with monoclonal antibodies as described (Hagman et al., 1989) and analyzed by FACScan (Becton-Dickinson). The following antibodies were used: phycoerythrin (PE) conjugated rat anti-mouse κ (Becton-Dickinson); biotinylated rat anti-mouse CD45R (clone RA3-6B2; PharMingen); and biotinylated rat anti-mouse λ (1+2) (clone R26-46; PharMingen), detected by subsequent staining with fluorescein isothiocynate (FITC)-conjugated streptavidin (Jackson ImmunoResearch). Spleen and bone marrow cells stained with anti-λ (1+2) were pretreated with purified rat anti-mouse Fcγ II receptor/CD32 (clone 2.4G2; PharMingen) at 0.6 μg and 0.3 μg/$10^6$ cells, respectively. Unless otherwise indicated, cells in the lymphocyte gate as defined by forward light scatter were analyzed.

### ACKNOWLEDGMENTS

We are grateful to T. Roth for help with the FACS analysis, M. Schlissel and H. Singh for primers and a hybridization probe to detect κ transcripts, and P. Engler for critical reading of the manuscript. This work was supported by NIH grant HD23089. Use of the Flow Cytometry Facility is partly supported by the University of Chicago Cancer Center Support Grant P30-CA14599. L. D. was supported by a fellowship from the National Multiple Sclerosis Society.

*(Received February 8, 1994)*

*(Accepted March 23, 1994)*

### REFERENCES

Alt F., Rosenberg N., Lewis S., Thomas E., and Baltimore D. (1981). Organization and reorganization of immunoglobulin genes in A-MuLV-transformed cells: Rearrangement of heavy but not light chain genes. Cell **27**: 381–390.

Alt F.W., Yancopoulos G.D., Blackwell T.K., Wood C., Thomas E., Boss M., Coffman R., Rosenberg N., Tonegawa S., and Baltimore D. (1984). Ordered rearrangement of immunoglobulin heavy chain variable region segments. EMBO **3**: 1209–1219.

Bogen B., and Weiss S. (1991). A rearranged $\lambda_2$ light chain retards but does not exclude κ and $\lambda_1$ expression. Eur. J. Immunol. **21**: 2391–2395.

Carson S., and Wu G. (1989). A linkage map of the mouse immunoglobulin lambda light chain locus. Immunogenetics **29**: 173–179.

Chomczynski P., and Sacchi N. (1987). Single-step method of RNA isolation by acid guanidinium thiocyanate-phenol-chloroform extraction. Analyt. Biochem. **162**: 156–159.

Coleclough C. (1983). Chance, necessity and antibody gene dynamics. Nature **303**: 23–26.

Coleclough C., Perry R.P., Karjalainen K., and Weigert M. (1981). Aberrant rearrangements contribute significantly to the allelic exclusion of immunoglobulin gene expression. Nature **290**: 372–378.

Cuisinier A., Fumoux F., Fougerau M., and Tonnelle C. (1992). IGMκ/λ EBV human B cell clone: An early step of differentiation of fetal B cells or a distinct B lineage? Mol. Immunol. **29**: 1363–1373.

Eccles S., Sarner N., Vidal M., Cox A., and Grosveld F. (1990). Enhancer sequences located 3′ of the mouse immunoglobulin λ locus specify high-level expression of an immunoglobulin λ gene in B cells of transgenic mice. New Biologist **2**: 801–811.

Eisen H., and Reilly E. (1985). Lambda chains and genes in inbred mice. Annu. Rev. Immunol. **3**: 337–365.

Eisenbeis C., Singh H., and Storb U. (1993). PU. 1 is a component of a multiprotein complex which binds an essential site in the murine immunoglobulin λ2-4 enhancer. Mol. Cell. Biol. **13**: 6452–6461.

Engler P., Weng A., and Storb U. (1993). Influence of CpG methylation and target spacing on V(D)J recombination in a transgenic substrate. Mol. Cell. Bio. **13**: 571–577.

Fort P., Piechaczyk M., Sabrouty S., Dani C., Jeanteur P., and Blanchard J. (1985). Various rat adult tissues express only one major mRNA species from the glyceraldehyde-3-phosphate-dehydrogenase multigene family. Nucl. Acids Res. **13**: 1431–1442.

Gollahon K.A., Hagman J., Brinster R.L., and Storb U. (1988). Ig λ-producing B cells do not show feedback inhibition of gene rearrangement. J. Immunol. **141**: 2771–2780.

Grey H., and Mannik M. (1965). Specificity of recombination of H and L chains from human γG myeloma proteins. J. Exp. Med. **122**: 619–630.

Hagman J. (1989). Murine immunoglobulin lambda genes are regulated by downstream enhancer elements. Ph.D. thesis, University of Washington.

Hagman J., Lo D., Doglio L.T., Hackett Jr. J., Rudin C.M., Haasch D., Brinster R., and Storb U. (1989). Inhibition of immunoglobulin gene rearrangement by the expression of a λ2 transgene. J. Exp. Med. **169**: 1911–1929.

Hagman J., Rudin C., Haasch D., Chaplin D., and Storb U. (1990). A novel enhancer in the immunoglobulin λ locus is duplicated and functionally independent of NFκB. Genes Devel. **4**: 978–992.

Hellman L., Steen M., Petterson U. (1985). Nonfunctional immunoglobulin light chain transcripts in two IgE-producing rat immunocytomas; implications for the allelic exclusion and transcription activation process. Gene **40**: 115–124.

Hieter P.A., Korsmeyer S. J., Waldmann T.A., and Leder P. (1981). Human immunoglobulin κ light-chain genes are deleted or rearranged in λ-production B cells. Nature **290**: 368–372.

Kim J.Y., Kurtz B., Huszar D., and Storb U. (1994). Crossing the SJL λ locus into κ-knockout mice reveals a dysfundtion of the λ1-containing immunoglobulin receptor in B cell differentiation. EMBO J. **13**: 827–834.

Klein M., Kostan C., Kells D., and Dorrington K. (1979). Equilibrium and kinetic aspects of the interaction of isolated variable and constant domains of light chain with the Fd′ fragment of immunoglobulin G. Biochemistry **18**: 1473–1481.

Lauster R., Reynaud C.A., Martensson I.L., Peter A., Bucchini D., Jami J. and Weill J.C. (1993). Promoter, enhancer, and silencer elements regulate rearrangement of an immunoglobulin transgene. EMBO J. **12**: 4615–4623.

Leclercq L., Butkeraitis P., and Reth M. (1989). A novel germ-line Jκ transcript starting immediately upstream of Jκ1. Nucl. Acids Res. **17**: 6809–6819.

Lewis M., Rosenberg N., Alt F., and Baltimore D. (1982). Continuing kappa gene rearrangement in a cell line transformed by Abelson murine leukemia virus. Cell **30**: 807–816.

Ma A., Fischer P., Dildrop R., Oltz E., Rathburn G., Achacoso P., Stall A., and Alt F.W. (1992). Surface IgM mediated regulation of RAG gene expression in Eμ-N-myc B cells lines. EMBO J. **11**: 2727–2734.

Manz J., Denis K., Witte O., Brinster R., and Storb U. (1988). Feedback inhibition of immunoglobulin gene rearrangement by membrane μ but not by secreted μ heavy chains. J. Exp. Med. **168**: 1363–1381.

Margulies D., Kuehl M., and Scharff M. (1976). Somatic cell hybridization of mouse myeloma cells. Cell **8**: 405–413.

Miller J., Ogden S., McMullen M., Andres H., and Storb U. (1988). The order and orientation of mouse λ-genes explain λ-rearrangement patterns. J. Immunol. **141**: 2497–2502.

Miller J., Selsing E., and Storb U. (1982). Structural alterations in J regions of mouse immunoglobulin λ genes are associated with differential gene expression. Nature **295**: 428–430.

Moore M., Durdik J., Persiani D., and Selsing E. (1985). Deletions of κ chain constant region genes in mouse λ chain producing B cells involve intrachromosomal DNA recombinations similar to V–J joining. Proc. Natl. Acad. Sci USA **82**: 6211–6215.

Neuberger M.S., Caskey H.M., Petterson S., Williams G.T., and Surani M.A. (1989). Isotype exclusion and transgene down-regulation in immunoglobulin-λ transgenic mice. Nature **338**: 350–352.

Picard D., and Schaffner W. (1984). A lymphocyte specific enhancer in the mouse immunoglobulin κ gene. Nature **307**: 80–82.

Pongubala J., Nagulapalli M., Klemsz S., McKercher S., Maki R., and Atchison M. (1992). PU.1 recruits a second nuclear factor to a site important for immunoglobulin κ 3′ enhancer activation. Mol. Cell. Biol. **12**: 368–378.

Ramsden D.A., and Wu G.E. (1991). Mouse kappa-light-chain recombination signal sequences mediate recombination more frequently than do those of lambda-light chain. Proc. Natl. Acad. Sci. **88**: 10721–10725.

Ritchie K.A., Brinster R.L., and Storb U. (1984). Allelic exclusion and control of endogenous immunoglobulin gene rearrangement in κ transgenic mice. Nature **312**: 517–520.

Rolink A., Grawunder U., Haasner D., Strasser A., and Melchers F. (1993). Immature surface $Ig^+$ B cells can continue to rearrange κ and λ chain gene loci. J. Exp. Med. **178**: 1263–1270.

Roth P., Doglio L., Manz J., Kim J.Y., Lo D., and Storb U. (1993). Immunoglobulin γ2b transgenes inhibit heavy chain gene rearrangement, but cannot promote B cell development. J. Exp. Med. **178**: 2007–2021.

Rudin C., Hackett J., and Storb U. (1991). Precursors of both conventional and Ly-1 B cells can escape feedback inhibition of Ig gene rearrangement. J. Immunol. **146**: 3205–3210.

Schlissel M.S., and Baltimore D. (1989). Activation of immunoglobulin kappa gene rearrangement correlates with induction of germline kappa gene transcription. Cell **58**: 1001–1007.

Storb U., Haasch D., Arp B., Sanchez P., Cazenave P., and Miller J. (1989). Physical linkage of mouse λ genes by pulsed-field gel electrophoresis suggests that the rearrangement process favors proximate target sequences. Mol. Cell. Biol. **9**: 711–718.

Strasser A., Rolink A., and Melchers F. (1989). One synchronous wave of B cell development in mouse fetal liver changes at day 16 of gestation from dependence to independence of a stromal cell environment. J. Exp. Med. **170**: 1973–1986.

Takeda S., Zou Y., Bluethman H., Kitamura D., Muller U., and Rajewsky K. (1993). Deletion of the immunoglobulin κ chain intron enhancer abolishes κ chain gene rearrangement in cis not not λ gene rearrangement in trans. EMBO J. **12**: 2392–2336.

Tiegs S., Russell D., and Nemazee D. (1993). Receptor editing in self-reactive bone marrow B cells. J. Exp. Med. **177**: 1009–1020.

Tonegawa S. (1983). Somatic generation of antibody diversity. Nature **302**: 575–581.

Tsang H., Pinkert C., Hagman J., Lostrum M., Brinster R.L., and Storb U. (1988). Cloning of a γ2b gene encoding anti-*Pseudomonas aeruginosa* H chains and its introduction into the germ line of mice. J. Immunol. **141**: 308–314.

Turka L., Schatz D., Oettinger M., Chun J., Gorka C., Lee K., McCormack W., and Thompson C. (1991). Thymocyte expression of RAG-1 and RAG-2: Termination by T cell receptor cross-linking. Science **253**: 778–781.

Weaver D., Costantini F., Imanishi-Kari T., and Baltimore D. (1985). A transgenic immunoglobulin mu gene prevents rearrangement of endogenous genes. Cell **42**: 117–127.

Wu G., Nusrat G., Hozumi N., and Murialdo H. (1982). Nucleotide sequence of a chromosomal rearranged λ2 immunoglobulin gene of mouse. Nucl. Acids Res. **10**: 3831–3843.

